# Associations between rapid weight gain in infancy and weight status among urban Aboriginal children participating in the Gudaga study: nine-year results from a cohort study

**DOI:** 10.1186/s12887-020-02121-w

**Published:** 2020-05-18

**Authors:** Elizabeth Denney-Wilson, Kaniz Fatema, Emma Elcombe, Suzanne Ingram, Mark Harris, Elizabeth Comino

**Affiliations:** 1 Faculty of Medicine and Health, The University of Sydney, Camperdown, New South Wales 2050 Australia; 2 Sydney Local Health District, Sydney, New South Wales Australia; 3 Centre for Health Equity Training, Research and Evaluation (CHETRE), part of the Centre for Primary Health Care and Equity, Faculty of Medicine, University of New South Wales, Sydney, New South Wales 2052 Australia; 4 Translational Research and Social Innovation, School of Nursing and Midwifery, Western Sydney University, Penrith, New South Wales 2751 Australia; 5 Centre for Primary Health Care and Equity, Faculty of Medicine, University of New South Wales, Sydney, New South Wales 2052 Australia

**Keywords:** Child obesity, Rapid weight gain, Aboriginal children, Body mass index, Waist to height ratio

## Abstract

**Background:**

Rapid weight gain (RWG) in infants is associated with overweight and obesity in childhood and beyond, highlighting the need for early intervention.

**Methods:**

Data from a birth cohort of Australian Aboriginal and Torres Strait Islander children living in an urban area were analysed to determine the prevalence of RWG in infancy and the association between RWG and overweight and obesity, categorised using both body mass index and waist to height ratio from birth to 9 years.

**Results:**

The prevalence of overweight and obesity is higher in this cohort (at 47%) than the population average. The Australian population as a whole has seen steady increases. In this cohort although the prevalence of combined overweight and obesity remained relatively stable between 2 and 9 years, the proportion of children categorized as obese using BMI has increased. 42% of children who were overweight or obese at 9 years had experienced RWG in infancy. Children were 2.7 and 3.9 times more likely to be overweight at 9 years if they experienced RWG or were overweight at 2 years, respectively.

**Conclusion:**

RWG was common in this cohort and the strongest predictor of excess weight at 2 years and at 9 years. Early intervention is crucial in the first year of life across the whole population to prevent obesity in children. Culturally appropriate interventions developed with the community are required for Aboriginal and Torres Strait Islander babies and their parents.

## Background

Recent data suggests that the population prevalence of combined overweight and obesity among Australian primary school aged children is relatively stable at 25%; however, substantial socioeconomic and cultural disparities exist and require attention [1]. In particular, children from low socioeconomic status backgrounds are twice as likely to be overweight or obese compared with their more advantaged peers [1, 2]. Of particular concern is that the prevalence of combined overweight and obesity among children from Aboriginal and Torres Strait Islander backgrounds is much higher than the population average, with data from the Australian Bureau of Statistics suggesting 40% of 10 year old children are either overweight or obese [3]. Aboriginal and Torres Strait Islander peoples hold distinct cultural identities whether they live in urban, regional or remote areas of Australia. The appropriate terminology referring to Aboriginal and Torres Strait Islander peoples is locally determined. Both Aboriginal and Torres Strait Islander people are included in the term ‘Aboriginal’ throughout this paper.

Improving the health outcomes for Aboriginal Australians has been a major priority of successive national and state governments during recent years. The Closing the Gap [4] strategy was introduced in 2008 to underline the importance of reducing health disparities and to coordinate activities and their funding. Targeting overweight and obesity will address one major risk factor for the health of Aboriginal Australians in childhood and in adult life. As a first step, it is important to explore the patterns of weight gain across childhood and to understand potential associations with weight gain in later life. This information is crucial to guide the development of culturally relevant and timely interventions for Aboriginal children, many of whom live in urban areas of Australia and may have different risk factors and environmental exposures from Aboriginal Australians living in rural and remote areas.

The Gudaga study included measurement of participant’s growth from 0 to 9 years. In this paper, we report on weight status, patterns of growth and explore the associations between rapid weight gain (RWG) and obesity at 9 years as categorised by both body mass index (BMI) and waist-to-height-ratio (WHtR).

## Methods

### Gudaga study summary

The population and methods of the Gudaga Study have been previously published [5]. Briefly, mothers of Aboriginal children were identified within the maternity ward of Campbelltown Hospital in outer urban Sydney, New South Wales, Australia, between May 2005 and October 2007 and recruited to the cohort. Participating parents agreed to data collection and to the extraction of their child’s data from available health service records. Interviews with mothers were undertaken at 2–3 weeks of age, and then at 6-monthly intervals from 6 months to 9 years of age. Anthropometric measures were collected at interview during the preschool years and then annually once children were at school. In total, 159 mothers and their children were identified at birth, and 149 were recruited at the first formal interview at 2–3 weeks of age.

### Anthropometry

Anthropometric measures included height, weight, head circumference and waist circumference (from 36 months of age). Height was measured to the nearest 0.1 cm in bare feet. Body weight was measured to the nearest 0.1 kg with a balance-beam scale with participants wearing lightweight clothing. BMI (kg/m^2^) was calculated as weight (kg) divided by the square of height (m). Waist circumference was measured to the nearest 0.1 cm at the mid-point between the lower costal border and the top of the iliac crest with the measurement taken at the end of a normal expiration. WHtR was calculated as waist divided by height.

### Final sample

Children who withdrew from the study prior to their 18 month interview (*n* = 27) were excluded from this analysis, as were children without anthropometric measures at birth or 12 months of age (*n* = 4). One hundred and twenty-eight children were included in this study.

#### Statistical analysis

All analyses were conducted using IBM SPSS V22.0 for Windows (Armonk, NY: IBM Corp). Continuous and categorical data were summarised using conventional summary statistics based on the mean measure with standard deviation (SD) for continuous variables, or proportion with the attribute of interest for categorical variables and their associated standard error. Logistic regression was used to calculate the relationship between selected risk factors and overweight or obesity at 9 years of age in both univariate and multivariate models. Variables with a statistical significance less than 0.2 in the univariate analysis were included in the multivariate model. The multivariate model used the backward stepwise method to consider the effect of selected risk factors.

Child weight, height (or length for children under 2 years of age), waist and BMI values were standardised using the Centre for Disease control (CDC) 2000 LMS method of standardisation [6]. The standardised measures were expressed as a z-score and percentile rank score.

The variables tested in the regression models included maternal factors (smoking, marital status, age, education, and a measure of socioeconomic status by suburb of residence, the Socio-Economic Indexes for Areas (SEIFA)); infant measures at birth (female child, low birth weight (< 2.5 kg), prematurity (less than 37 weeks gestation), and small for gestational age (SGA)), and infant measures during the first 12 months of life (breastfeeding and RWG). RWG was defined as an increase in weight-for-age SD score greater than or equal to 0.67 between birth and 12 months of age [6].

#### Ethics

Signed informed consent was obtained from the mothers of all participating children at birth, 12 months and 5 years. The study has the cooperation and support of the local Aboriginal community [5, 7].

This study was approved by the ethics committees of the Aboriginal Health and Medical Research Council (679/11) and the South West Sydney Local Health District (HREC/10/LPOOL/202).

## Results

### Cohort description

Data for this paper was available for 128 Aboriginal children. The full cohort includes 149 child/parent dyads and the characteristics of the dyads included in this paper did not differ significantly from the full cohort (data not shown). At time of birth, mothers of Aboriginal infants were on average, aged 25.6 years (SD = 6.53); 23% were aged less than 20 years of age; 42% were single; 55% lived in a suburb classified within the lowest SEIFA quintile; 25% had not completed year 10 of high school; and more than 50% of mothers smoked during their pregnancy. Low rates of breastfeeding were reported, with 65% of Aboriginal infants receiving any breast milk and only 13% being partly or completely breastfed at 20 weeks. At birth, 7% of infants weighed less than 2.5 kg, 8% were born premature (less than 37 weeks gestation), mean gestation length was 39.4 weeks (SD = 1.8) and 12.5% were considered SGA.

### Patterns of growth

**Fig. 1:**
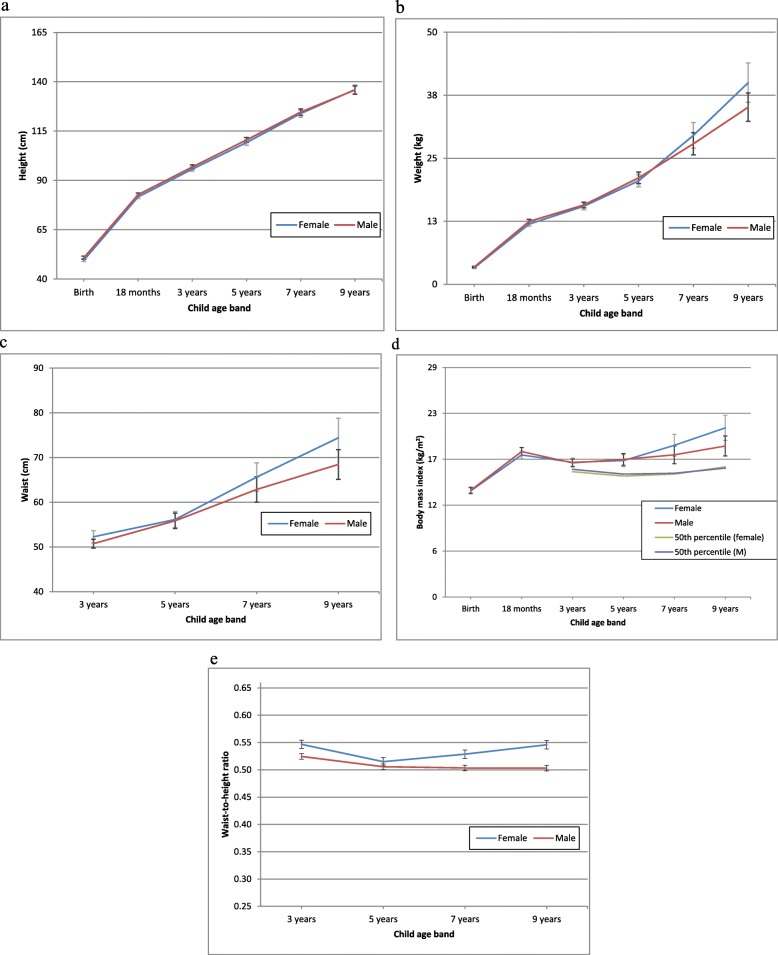
Patterns of growth for Gudaga children from birth to 9 years of age **a**: Mean height (cm) together with 95% confidence intervals **b**: Mean weight (kg) together with 95% confidence intervals **c**: Mean waist (cm) together with 95% confidence intervals **d**: Mean body mass index together with 95% confidence intervals and the median BMI, or the 50th percentile, at each age according to the CDC 2000 data set. **e**: Mean waist-to-height ratio together with 95% confidence intervals

The patterns of growth (height, weight and waist) are summarised in Fig. 1 (a, b, c, d and e). At birth, infants averaged 50.2 cm (SD = 3.02) in length and 3.4 kg (SD = 0.56) in weight. When infant weight was standardised, the mean standardised weight-for-age z-score at birth was − 0.22. At 3 years of age, average child height was 96.2 cm (SD = 4.37) and weight was 15.6 kg (SD = 2.43); and the mean weight-for-age z-score had risen over half a SD to be 0.52 above the CDC standard. At 9 years of age, average child height was 136.0 cm (SD = 7.2), and weight was 37.8 kg (SD = 11.5); and the mean standardised weight-for-age z-score was 0.69 SD above the CDC Standard.

### Growth measurement standardisation and percentile banding

**Fig. 2:**
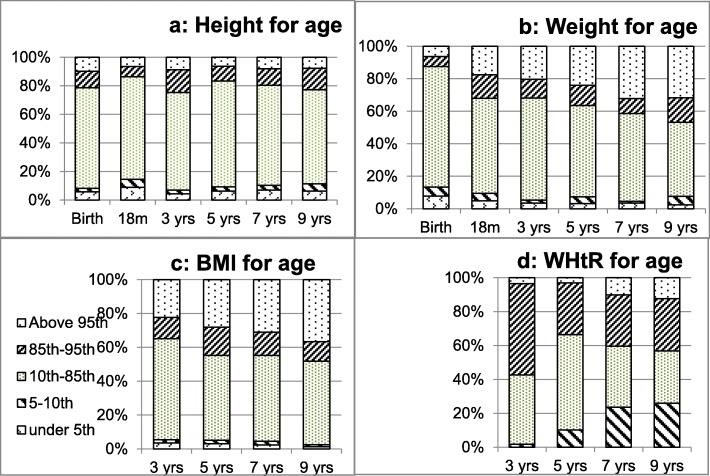
Children within each age standardised percentile band from birth to 9 years of age. **a**: Height for age **b**: Weight for age **c**: BMI for age **d**: Waist-to-height ratio for ageBody mass index

The age standardised percentiles for children’s growth are shown in Fig. 2. The majority of Aboriginal children at each age were within normal height (Fig. 2 a) and weight (Fig. 2 b) ranges (10th–85th percentile), as 66–74% and 46–74% respectively.

Using the BMI z-score categorisation, 34% of the cohort were categorised as being overweight or obese (85th percentile and above) at 3 years. Just over 50% of children at 5, 7 and 9 years of age had a healthy BMI (10th–85th percentile) compared to the target percentage of 75% (Fig. 2 b). The proportion of overweight and obese children rose to 45% by 5 years of age, then to 48% by 9 years of age.

### Waist-to-height ratio or waist-to-stature ratio

Using WHtR categorisation, there are no extremely slim children in the ≤5th percentile and only 1.7% of children were slim (5th–10th percentile) at the age of 3 years. Almost 41% were within the optimum healthy range (10th–85th percentile) and 57.4% of the cohort were recorded as being ‘overweight’ or ‘very obese’ (85th percentile and above) at 3 years of age. The proportion of ‘very slim and slim’ (10th percentile and below) and ‘overweight and obese’ children rose to 10.2 and 33.7% respectively by 5 years of age, then to 23.6 and 40.4% by 7 years, and 25.9 and 43.2% by 9 years of age. At the ages of 7 and 9 years, only 36 and 31% of the cohort fell into the healthy band of WHtR (Fig. 2 d).

### Associations between rapid weight gain and overweight and obesity

At 9 years of age, 37 children (47%) of the cohort had a BMI-for-age above the 85th percentile. At 2 years of age, RWG in the first 12 months was significantly associated with overweight and obesity. RWG remained significantly associated with overweight and obesity at 9 years of age (*p* < 0.006). In addition to RWG, having a BMI over the 85th percentile at 2 years of age was associated with overweight and obesity at 9 years of age (*p* < 0.002) The variables tested in the regression models are shown in Table 1.

**Table 1:** Risk factors for overweight and obesity at 9 years of age among Aboriginal children participating in the Gudaga Study

	Demographics		Univariate (*n* = 79)		Multivariate		
	Birth n (%)	9 years n (%)	Exp (β)	*p*-value		Exp (β)	*p*-value
Mother single	74 (58%)	45 (57%)	0.71	0.455			
Mother aged < 20 years	29 (23%)	10 (13%)	0.69	0.585			
Low maternal education	32 (25%)	17 (22%)	0.95	0.923			
Lowest SEIFA	70 (55%)	47 (60%)	0.58	0.233			
Female infant	68 (53%)	44 (56%)	**1.80**	**0.200**	✓	–	ns
Low infant birth weight (< 2500 g)	9 (7%)	5 (6%)	0.25	0.225			
Premature infant (< 37 weeks)	10 (8%)	6 (8%)	0.51	0.458			
SGA infant	16 (13%)	10 (13%)	1.73	0.424			
Maternal smoking during pregnancy	77 (61%)	48 (61%)	2.34	0.074	✓	2.564	0.082
Breastfeeding at discharge	68 (53%)	42 (53%)	0.78	0.588			
Breastfeeding for > 20 weeks	17 (13%)	8 (10%)	1.62	0.530			
RWG (0–12 months)	43 (34%)	33 (42%)	**3.75**	**0.006**	✓	3.241	0.034
BMI >85th at 24 months^a^	45 (37%)	30 (40%)	**4.82**	**0.002**	✓	3.157	0.037

*BMI* Body mass index

Exp(B): estimate for fixed effect: represents of odds of the child being overweight or obese when the risk factor is present, compared to when the risk is not present

*RWG* Rapid weight gain

*SEIFA* Socio-economic indexes for areas

Low Education - did not complete year 10

*SGA* Small for gestational age

^a^ Measure missing for 3 participants

✓ = item included in multivariate model

In the combined model, children who experienced RWG were 2.7 times more likely to be overweight at 9 years than children who did not experience RWG. Similarly, children who had a BMI over the 85th percentile at 2 years were 3.9 times more likely to be overweight at 9 years of age, compared to children who were not overweight at 2 years of age. Female children were 2.4 times more likely to be overweight at 9 years of age, than males. None of the other factors tested in the model achieved significance.

## Discussion

This study aimed to describe the growth trajectories of Australian Aboriginal children from 0 to 9 years within an urban setting and to determine whether children who experienced RWG and were overweight or obese at 2 years remain above a healthy weight at 9 years of age. Just over half the Gudaga children were in the healthy weight range at each time point up to 9 years. The prevalence of combined overweight and obesity is higher than the population average, and has remained relatively stable over time. However the proportion of children in the obese range has increased. Similarly, data from the New South Wales Schools Physical Activity and Nutrition Survey suggested that among children from low socioeconomic backgrounds, the prevalence of combined overweight and obesity had increased from 29.1% in 2010 to 33.7% in 2015 [1].

At 9 years of age, factors from early life: RWG and having a BMI above the 85th centile at 2 years of age, were the most significant predictors of overweight and obesity among the Gudaga cohort. RWG has been consistently associated with child and adult obesity with a recent meta-analysis of 17 studies finding that infants who experienced RWG were at 3.66 times (2.59–5.17, 95% CI) greater odds of being overweight or obese later in life (from ages 2 to 46.5 years), than those who did not experience RWG [8].

Infants fed with formula are more likely to experience RWG [9] with data from an Australian cohort finding that the only two modifiable risk factors associated with RWG were formula feeding and feeding to a schedule. In the Gudaga cohort both initiation and duration of breastfeeding were very low, with only 53% of mother’s initiating breastfeeding and only 10% still breastfeeding at 20 weeks. Therefore, formula feeding was more common in this cohort than in the general population and may explain some of the RWG. Both the content of formula (some studies suggest that formula with a higher protein content is associated with RWG) [10] and feeding practices may be implicated.

A recent review has found that some infant formula feeding practices are associated with RWG [11] such as higher volumes of formula, pressure to finish a bottle and using a bottle to put an infant to bed. This suggests that interventions aimed at increasing the duration of breastfeeding and promoting formula feeding practices such as providing formula with lower protein content, not putting a baby to bed with a bottle and not overfeeding formula may be important prevention strategies for all children [9].

**Fig. 3:**
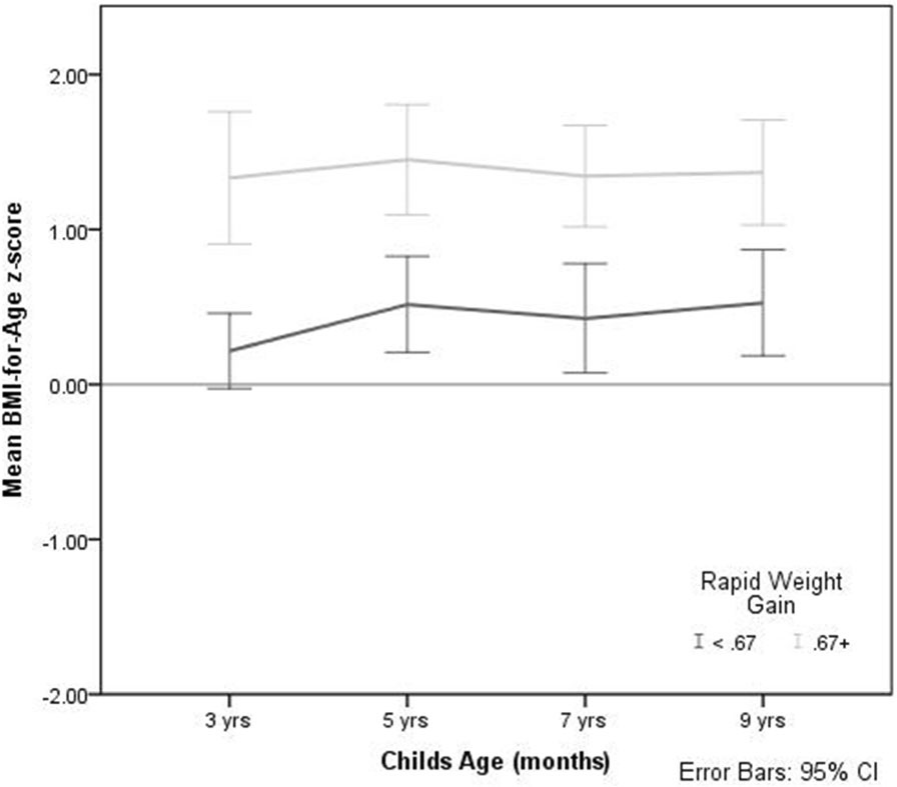
BMI z-score by RWG

This study adds to the evidence that early intervention, in the first year of life is crucial in tackling child and adult obesity. Preventing RWG in infancy through strategies such as promoting breastfeeding and providing support for parents who are formula feeding to ensure that overfeeding does not occur are urgently needed for all families (Fig. 3).

### Strengths and limitations

While our sample size is relatively small, this is, to our knowledge, the first study to provide detailed longitudinal growth data from a cohort of Australian Aboriginal children living in an urban area in Sydney. We did not explore the impact of genetic or environmental factors on the high rates of overweight and obesity and RWG, and we do not have reliable information on the antenatal weight gain or care of the mothers. We did not examine the perception of infant growth among Gudaga parents or the local Aboriginal community.

The consistency of the study team including a local Aboriginal woman conducting data collection is a key strength of the study.

## Conclusion

This study found high rates of overweight and obesity at 9 years of age in a cohort of Aboriginal children in Sydney, Australia. RWG in the first year of life and being overweight at 2 years were associated with obesity at 9 years. RWG is a modifiable risk factor for obesity and should be targeted by health services through interventions that are tailored to infant feeding method and are culturally appropriate.

## Data Availability

The datasets generated and/or analysed during the current study are not publicly available due to data sharing stipulations in the ethics application but are can be requested from the corresponding author and the community custodians.

## Abbreviations

BMI: Body mass index
CDC: Centers for disease control and prevention
RWG: Rapid weight gain
SD: Standard deviation
SEIFA: Socio-economic indexes for areas
SGA: Small for gestational age
WHtR: Waist-to-height ratio
